# Chromosomal radiosensitivity in G_2_-phase lymphocytes identifies breast cancer patients with distinctive tumour characteristics

**DOI:** 10.1054/bjoc.2001.2086

**Published:** 2001-10

**Authors:** A C Riches, P E Bryant, C M Steel, A Gleig, A J Robertson, P E Preece, A M Thompson

**Affiliations:** Medical Science and Human Biology, School of Biology, University of St Andrews, St Andrews, KY16 9TS; Department of Molecular and Cellular Pathology, University of Dundee; Department of Surgery and Molecular Oncology, University of Dundee, Dundee, DD1 9SY

**Keywords:** breast cancer, chromosomal radiosensitivity, G_2_-phase

## Abstract

A substantial proportion of women with breast cancer exhibit an abnormally high radiosensitivity as measured by the frequency of chromatid breaks induced in G_2_-phase, PHA stimulated lymphocytes. Chromatid break frequencies were compared for a cohort of previously untreated sporadic breast cancer patients and hospital outpatient controls. In the breast cancer group 46% showed high radiosensitivity compared to 14% of controls (*P*< 0.001). Comparison of those breast cancer patients with a high G_2_radiosensitivity (G_2_RS) versus those with a low G_2_RS showed no difference in menopausal status or age but the high G_2_RS group had on average a lower score on the Nottingham Prognostic Index. Predicted survival in the high G_2_RS group at 15 years was 55% compared to 36% for the low G_2_RS group. Furthermore, 81% of tumours from the high G_2_RS were oestrogen receptor positive compared to 45% from the low G_2_RS group. Thus high G_2_RS identifies a sub-population of patients with distinctive tumour characteristics and with a predicted improved prognosis as compared with those in the low G_2_RS group. Our findings imply that besides influencing risk of breast cancer the genetic factors determining G_2_radiosensitivity also influence the tumour characteristics and prognosis in these patients. © 2001 Cancer Research Campaign  http://www.bjcancer.com


*British Journal of Cancer* (2001) **85**(8), 1157–1161
© 2001 Cancer Research Campaign
doi: 10.1054/ bjoc.2001.2086, available online at http://www.idealibrary.com on IDEAL

### Chromosomal radiosensitivity in $G_2$-phase lymphocytes identifies breast cancer patients with distinctive tumour characteristics

**AC Riches[1], PE Bryant[1], CM Steel[1], A Gleig[1], AJ Robertson[2], PE Preece[3] and AM Thompson[3]**

[1]Medical Science and Human Biology, School of Biology, University of St Andrews, St Andrews KY16 9TS; [2]Department of Molecular and Cellular Pathology, University of Dundee; and [3]Department of Surgery and Molecular Oncology, University of Dundee, Dundee DD1 9SY

**Summary** A substantial proportion of women with breast cancer exhibit an abnormally high radiosensitivity as measured by the frequency of chromatid breaks induced in $G_2$-phase, PHA stimulated lymphocytes. Chromatid break frequencies were compared for a cohort of previously untreated sporadic breast cancer patients and hospital outpatient controls. In the breast cancer group 46% showed high radiosensitivity compared to 14% of controls ($P < 0.001$). Comparison of those breast cancer patients with a high $G_2$ radiosensitivity ($G_2$RS) versus those with a low $G_2$RS showed no difference in menopausal status or age but the high $G_2$RS group had on average a lower score on the Nottingham Prognostic Index. Predicted survival in the high $G_2$RS group at 15 years was 55% compared to 36% for the low $G_2$RS group. Furthermore, 81% of tumours from the high $G_2$RS were oestrogen receptor positive compared to 45% from the low $G_2$RS group. Thus high $G_2$RS identifies a sub-population of patients with distinctive tumour characteristics and with a predicted improved prognosis as compared with those in the low $G_2$RS group. Our findings imply that besides influencing risk of breast cancer the genetic factors determining $G_2$ radiosensitivity also influence the tumour characteristics and prognosis in these patients. © 2001 Cancer Research Campaign http://www.bjcancer.com

**Keywords**: breast cancer; chromosomal radiosensitivity; $G_2$-phase

The association between chromosomal radiosensitivity and cancer predisposition has been clearly demonstrated in a number of heritable conditions (Sanford et al, 1989). This was first established for patients with the recessively-inherited multi-system disorder ataxia-telangiectasia (A-T) (Taylor, 1983) and further demonstrated in 20 other inherited cancer-prone conditions (Scott et al, 1996, 1999).

Scott et al (1994) reported that, using peripheral blood lymphocytes stimulated with phytohaemagglutinin (PHA) in vitro, they could discriminate between A-T heterozygotes and normal controls according to the number of chromatid aberrations induced by low doses of radiation (the $G_2$ assay). They demonstrated that 42% of untreated breast cancer patients but only 9% of controls exhibit a $G_2$ sensitivity similar to that of A-T heterozygotes (Scott et al, 1994, 1998). Three other groups have confirmed these findings in breast cancer patients (Parshad et al, 1996; Patel et al, 1997; Terzoudi et al, 2000).

Increased chromosomal radiosensitivity among blood relatives of breast cancer patients with high $G_2$ scores have also been demonstrated. Clear evidence of Mendelian heritability of $G_2$ chromosomal radiosensitivity was established by Roberts et al (1999). A single major gene could account for 82% of the variance between family members. This strongly supports the view that enhanced radiosensitivity of peripheral blood lymphocytes is a marker for breast cancer-predisposing genes of limited penetrance.

*Received 14 May 2001*
*Accepted without revision 21 August 2001*

*Correspondence to:* AC Riches

It is well established that breast cancers arising in carriers of the breast cancer susceptibility genes, *BRCA1* and *BRCA2* have different histological characteristics from each other and from breast cancers arising in patients unselected for family history (Lakhani et al, 1997; 1998). A substantial proportion of families with multiple breast cancers, however, are not attributable to these two genes *BRCA1* and *BRCA2* (Peto et al, 1999). The pathological characteristics of tumours arising in non-*BRCA1* or non-*BRCA2* families are different from *BRCA1* and *BRCA2* tumours (Lakhani et al, 2000). There is also evidence that the pathological characteristics of non *BRCA1*/*BRCA2* familial breast cancers differ from sporadic (nonfamilial) breast cancers (Lakhani et al, 2000).

In breast cancer, a reliable indicator of prognosis has been developed, the Nottingham Prognostic Index (Galea et al, 1992), using information on tumour size, histological grade and nodal status (see Table 2 for formula).

No studies have compared the characteristics of breast cancers in groups identified by their $G_2$RS. We have therefore undertaken such a study.

### MATERIALS AND METHODS

### Patients blood samples and clinical data

Peripheral venous blood was collected from 65 unselected hospital outpatients with primary breast cancer at the time of attendance for diagnosis and from 66 control surgical outpatients without malignancy. The blood samples (10 ml) were collected into lithium heparin tubes and transported at room temperature by car to the St Andrews laboratory (a distance of 20 miles). Age, menstrual status, tumour size, histological nodal status, histopathological classification of tumour, histological grade and oestrogen receptor status were documented retrospectively for the breast cancer patients. Pathological assessment of tumours and oestrogen receptor status, recorded by standard protocols by a specialist breast pathologist, were undertaken independently of the $G_2$ assay. Results were collated after chromatid breaks were scored and were not known to those carrying out the $G_2$ assay.

All of the studies were undertaken with ethical approval from the Tayside and Fife Committees on Medical Research Ethics (218/97 and DE/JRW20069717).

### $G_2$ assay

The blood samples were held in the laboratory overnight at room temperature and set up for culture the following day. Two samples were set up from each subject by adding 1 ml of blood to 9 ml prewarmed and gassed medium (RPMI 1640 containing 10% FCS, l-glutamine and antibiotics) to a 25 $cm^3$ flask. PHA (Murex HA15, 150 μl of the 5 ml stock) was added to each sample. The flasks were placed flat in a humidified and gassed incubator (5% $CO_2$/95% air) for 72 h.

At this time one flask was exposed to 0.4 Gy gamma irradiation from a caesium 137 gamma source (IBL437C). The other sample was mock irradiated. After 30 min incubation, colcemid (150 μl of 10 μg/ml stock) was added to each flask and returned to the incubator for a further 1 h. The cells and medium were then transferred to a 10 ml centrifuge tube and held on ice for 10 min. Cells were treated with ice-cold hypotonic solution (0.075M KCI) for 10 min, fixed and metaphase preparations made using standard protocols. Slides were coded for scoring blind and 50–100 metaphase spreads scored for aberrations under 63× oil immersion lens. The number of chromatid breaks per 100 metaphases was determined: the '$G_2$ score'.

### Statistical analysis

For comparisons between the $G_2$ scores in breast cancer patients and controls, the Mann-Whitney test was applied. The proportions of patients and controls in the high $G_2$RS group were estimated using the 90th percentile of the control group as the cut-off, as described by Scott et al (1999). Differences in the proportions in this high $G_2$RS cohort were compared for the breast cancer patients and controls using the two-tailed Fisher's exact test. Two groups of breast cancer patients were further selected on the basis of their $G_2$ lymphocyte scores; 21 patients with the highest and 20 with the lowest $G_2$ scores. Comparisons between the patients in the high $G_2$RS group and the low $G_2$RS group for oestrogen receptor positivity, nodal involvement and menopausal status were undertaken using a $\chi^2$ test.

Comparisons of the median age and tumour diameter in these two groups used the Mann-Whitney test.

### RESULTS

### Reproducibility of the $G_2$ assay

Independent repeat assays on 7 donors allowed an estimate of the $G_2$ assay reproducibility (intra-individual variance) which gave a coefficient of variation (CV) of 8% compared with a CV of 19% for inter-individual differences between donors. Assays on blood collected at St Andrews and Dundee from the same donors on the same day enabled samples to be checked for possible differences introduced by the transport procedure. No differences were detected. The correlation coefficient was 0.76 ($P < 0.05$).

### Comparison of the $G_2$ scores in breast cancer patients and hospital controls

Figure 1 illustrates the distribution of $G_2$ scores for patients and controls. The mean overall $G_2$ score for the breast cancer patients (84.8 ± 36.8) was greater than that for the control group (63.3 ± 18.5) (Table 1, $P < 0.0001$). The median values were significantly different ($P < 0.0001$). Using the 90th percentile of the control group as the cut-off point, the proportion of breast cancer patients with high $G_2$ sensitivity was 46.2% compared to 13.6% for the controls (since scores were in bins, 13.6% approximates the closest to the 90th percentile). These proportions were significantly different ($P < 0.001$) indicating that there was a substantial sub-group of breast cancer patients exhibiting increased lymphocyte radiation sensitivity (Table 1).

### Comparison of the Nottingham Prognostic Index in breast cancer patients exhibiting high or low $G_2$ scores

For the breast cancer patients, those in the highest and lowest tertiles of $G_2$, scores were grouped using the 3 lowest and the 7

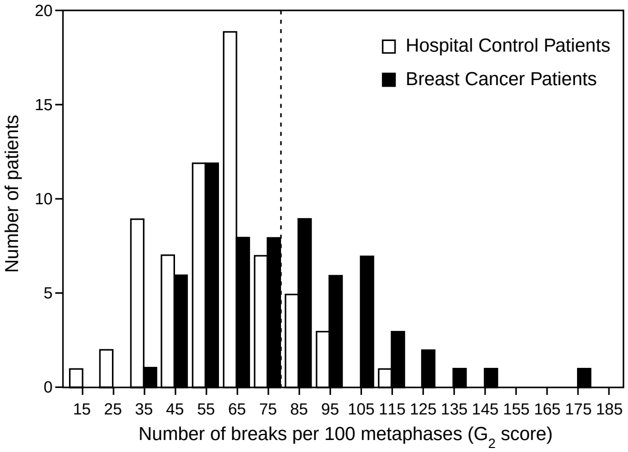

**Figure 1** Comparison of the $G_2$ scores (number of chromatid breaks per 100 metaphases) measured in irradiated peripheral blood lymphocytes from breast cancer patients and hospital outpatient controls (........90th percentile cut-off from the control population)

**Table 1** Mean $G_2$ scores (number of chromatid aberrations per 100 metaphases scored in peripheral blood lymphocytes), population standard deviations, ranges and proportions of sensitive breast cancer patients and normals in the $G_2$ assay

| | Normals | Breast cancer patients | |
|---|---|---|---|
| Mean $G_2$ score ± SD | 63.6 ± 18.5 | 84.8 ± 26.8 | $P < 0.0001$[a] |
| Range | 24 – 118 | 42 – 181 | |
| Sensitive sub-group | 9/66 (13.6%)[c] | 30/65 (46.2%)[c] | $P < 0.001$[b] |
| Number of subjects | 66 | 65 | |
| Median age | 47 | 58 | |
| Range | 20 – 76 | 29 – 95 | |

[a] Mann-Whitney U test; [b] Two-tailed Fisher's exact test; [c] The 90th percentile was selected as the cut-off (as results were grouped in bins, more than 10% of patients had scores equal to, or greater than, this figure).

 *© 2001 Cancer Research Campaign*

**Table 2** Comparison of the age, $G_2$ score, tumour diameter, stage, grade and the Nottingham Prognostic Index for two cohorts of breast cancer patients with a high or a low $G_2$ score

| | Age (years) | $G_2$ score | Tumour diameter (cm) | Stage | Grade | Nottingham Prognostic Index[a] |
|---|---|---|---|---|---|---|
| Low $G_2$ scores | 53 | 47 | 2.2 | 2 | 3 | 5.44 |
| | 45 | 46 | 3.0 | 2 | 3 | 5.60 |
| | 58 | 52 | 1.5 | 1 | 1 | 2.30 |
| | 50 | 42 | 4.5 | 2 | 2 | 4.90 |
| | 55 | 50 | 2.5 | 2 | 3 | 5.50 |
| | 32 | 54 | 1.8 | 1 | 3 | 4.36 |
| | 29 | 58 | 2.0 | 1 | 3 | 4.40 |
| | 57 | 63 | 2.5 | 1 | 2 | 3.50 |
| | 41 | 62 | 1.3 | 2 | 2 | 4.26 |
| | 60 | 64 | 3.1 | 1 | 2 | 3.62 |
| | 50 | 64 | 2.0 | 1 | 3 | 4.40 |
| | 69 | 66 | 2.3 | 2 | 2 | 4.46 |
| | 77 | 64 | 2.7 | 3 | 3 | 6.54 |
| | 55 | 62 | 2.0 | 1 | 2 | 3.40 |
| | 64 | 60 | 1.2 | 1 | 2 | 3.24 |
| | 60 | 64 | 3.6 | 3 | 3 | 6.72 |
| | 69 | 62 | 3.5 | 3 | 3 | 6.70 |
| | 55 | 60 | 2.2 | 3 | 3 | 6.44 |
| | 67 | 66 | 2.6 | 1 | 2 | 3.52 |
| | 84 | 56 | 3.0 | 2 | 3 | 5.60 |
| High $G_2$ scores | 60 | 181 | 0.8 | 2 | 2 | 4.16 |
| | 66 | 115 | 1.5 | 2 | 3 | 5.30 |
| | 77 | 113 | 1.8 | 1 | 2 | 3.36 |
| | 62 | 120 | 1.4 | 1 | 2.5 | 3.76 |
| | 49 | 106 | 2.0 | 1 | 2 | 3.40 |
| | 58 | 114 | 1.5 | 1 | 3 | 4.30 |
| | 51 | 126 | 1.0 | 1 | 2 | 3.20 |
| | 60 | 107 | 2.0 | 3 | 2 | 5.40 |
| | 53 | 125 | 1.1 | 1 | 3 | 4.22 |
| | 70 | 105 | 1.5 | 1 | 3 | 4.30 |
| | 53 | 101 | 1.7 | 2 | 1 | 3.34 |
| | 37 | 116 | 2.0 | 2 | 3 | 5.40 |
| | 35 | 110 | 2.0 | 2 | 1 | 3.40 |
| | 60 | 96 | 2.1 | 2 | 2 | 4.42 |
| | 56 | 96 | 1.2 | 1 | 1.5 | 2.74 |
| | 82 | 92 | 1.5 | 1 | 3 | 4.30 |
| | 95 | 92 | 2.8 | 1 | 2 | 3.56 |
| | 83 | 92 | 1.4 | 1 | 2 | 3.28 |
| | 54 | 138 | 1.7 | 1 | 2 | 3.34 |
| | 73 | 104 | 1.6 | 3 | 2 | 5.32 |
| | 59 | 112 | 1.5 | 3 | 3 | 6.30 |

[a] The Nottingham Prognostic Index (Galea et al, 1992) = histological grade + stage + (tumour diameter cm $\times$ 0.2).

highest blocks of scores from the histogram (Figure 1). The Nottingham Prognostic Index (NPI) was calculated using data on the size of the primary tumour, axillary lymph node status and histological grade (Table 2). The 15-year survival is related to the NPI and can be used to define three sub-groups; a good prognosis group (GPG: NPI < 3.4) with an 80% 15-year survival, a medium prognosis group (MPG: NPI 3.4–5.4) with a 42% 15-year survival and a poor prognosis group (PPG: NPI > 5.4) with a 13% 15-year survival. In the Nottingham study (Galea et al, 1992), the proportions of patients presenting in each sub-group were GPG 29%, MPG 54% and PPG 17%. In our total unselected Tayside cohort, the proportions were similar; GPG 27%, MPG 51% and PPG 22% ($\chi^2$ = 0.65; $P$ ~ 0.5) (Figure 2). However, when the breast cancer patients were separated into high and low $G_2$RS groups, the proportions changed markedly; GPG 15%, MPG 45% and PPG 40% in the low $G_2$RS group compared to GPG 38%, MPG 57% and PPG 5% in the high $G_2$RS group (Figure 2). In both cases these proportions were significantly different from those of the total unselected cohort (low $G_2$, $\chi^2$ = 4.13, $P$ < 0.05; high $G_2$, $\chi^2$ = 3.98, $P$ < 0.05).

### Comparison of the tumours and patients in the high and low $G_2$RS groups

The tumours observed in the low $G_2$RS group were all invasive ductal carcinomas whereas in the high $G_2$RS group there were two lobular carcinomas and one tubulo-lobular type. There were no significant differences between the low and high $G_2$RS groups in the median ages of the patients ($P$ ~ 0.3), the menopausal status ($\chi^2$ = 1.2, $P$ > 0.1) and the histological nodal status ($\chi^2$ = 0.22, $P$ > 0.5) (Table 3).

Both the median tumour diameter ($P$ < 0.001) and the proportion of oestrogen receptor positive tumours ($P$ < 0.05) were significantly different in the two groups. The low $G_2$RS group tended to have larger tumours which were oestrogen receptor negative

*© 2001 Cancer Research Campaign*

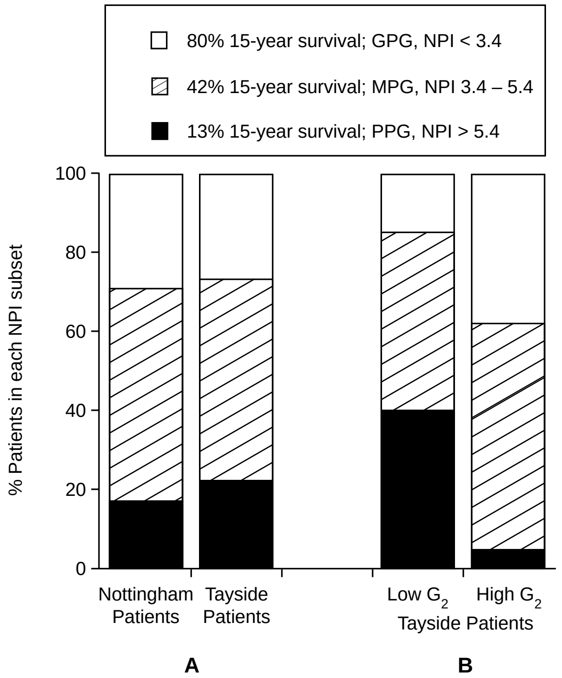

**Figure 2** Comparison of the percentage of breast cancer patients in each category defined by the Nottingham Prognostic Index (Galea et al, 1992). (**A**) Comparison of unselected breast cancer patients from Nottingham and Tayside. (**B**) Breast cancer patients from the Tayside group selected on the basis of high and low $G_2$ score whereas the high $G_2$RS group had tumours that were smaller and oestrogen receptor positive (Table 3).

A predicted 15-year survival can be calculated from the proportions of patients in each sub-group as indicated by the NPI. For the high $G_2$RS group this was 55% compared to 36% for the low $G_2$RS group. For the total unselected cohort, the predicted 15-year survival would be 46% (Table 3).

### DISCUSSION

We have confirmed that a significant proportion of breast cancer patients exhibit an elevated $G_2$RS value relative to controls. The proportion of radiosensitive patients observed in the Tayside cohort (46.2%) was similar to that reported in the Manchester studies (42%, Scott et al, 1994; 39% Scott et al, 1999; $\chi^2$ corr = 0.6, $P \sim 0.4$). These figures are consistent with data from epidemiological studies suggesting a substantial frequency of low penetrance genetic predisposition to breast cancer (Teare et al, 1994; Chen et al, 1995; Houlston and Peto, 1996). Increased chromosomal radiosensitivity has also been reported in breast cancer patients by Patel et al (1997), Terzoudi et al (2000) and Parshad et al (1996) although the numbers studied in these series do not permit close comparison of the proportions showing increased radiosensitivity.

Assay reproducibility between and within donor groups was similar to that previously reported (Scott et al, 1999). Problems with transport of blood samples were not encountered presumably because of the proximity of the centres in this study.

High lymphocyte $G_2$RS appears to be unrelated to sex, age or to environmental variables. There is now clear evidence that radiosensitivity, as measured by the $G_2$ assay, in breast cancer patients is inherited and likely to be a marker for a small number of low penetrance genes predisposing to breast cancer (Roberts et al, 1999). Thus from our data and those of Scott et al (1994;1999), women with a high $G_2$RS genotype are at approximately five-fold greater risk than those with a normal $G_2$RS: i.e. penetrance of this trait with respect to breast cancer is estimated at approximately 35%. Hence this heritable characteristic accounts for some 30% of all breast cancer cases, at least six times as many as all other currently recognized forms of heritable breast cancer combined. Patients in this study were not selected on the basis of family history and, given the estimated low penetrance of the cancer trait, detailed analysis of extended family trees would be required to demonstrate heritability.

The Nottingham Prognostic Index has proved useful in predicting survival in breast cancer patients and has been assessed prospectively (Galea et al, 1992; Brown et al, 1993; Balslev et al, 1994). The proportions of the three prognostic sub-groups in our cohort of unselected breast cancer patients were similar to those reported in the much larger study from Nottingham (Galea et al, 1992). However, when subdivided on the basis of the $G_2$ score, the results were quite different and patients in the low $G_2$RS group had a much poorer predicted prognosis than those in the high $G_2$RS group. This difference could not be explained on the basis of age or menopausal status. In fact rather more patients in the low $G_2$RS group were post-menopausal.

Several properties of the tumours in the low and high $G_2$RS groups showed distinct differences. In the high $G_2$ sensitive group, the tumours tended to be not only smaller at presentation but also oestrogen receptor positive. This would lead to a further improvement in prognosis for the high $G_2$RS group, compared to the low $G_2$RS group. Taking into account that the 15-year survival in age matched cancer-free controls is 83% then a predicted improvement from 36% to 55% 15-year survival in the low versus the high $G_2$RS group represents a dramatic difference.

**Table 3** Characteristics of patients and tumours in the high $G_2$ sensitivity group and the low $G_2$ sensitivity group

| | **High $G_2$[a] sensitivity** *n* = 21 | **Low $G_2$[a] sensitivity** *n* = 20 | |
|---|---|---|---|
| Median age (range) | 60 (30–95) | 56 (29–84) | $P$ ~0.3[b] |
| Mean $G_2$ score (breaks per 100 metaphases) | 112 ± 4 | 58 ± 2 | |
| Oestrogen receptor[c] % +ve | 81 | 45 | $P < 0.05$[d] |
| Median tumour diameter (cm) | 1.5 | 2.4 | $P < 0.001$[b] |
| Histological nodal status (% +ve) | 43 | 55 | $P > 0.5$[c] |
| Menopausal status (% postmenopausal) | 65 | 85 | $P > 0.1$[c] |
| Predicted 15-year survival (%) | 55 | 36 | |

[a]Highest and lowest tertile of the observed range; [b]Mann-Whitney U test; [c]Oestrogen receptor status defined using standard immunocytochemical methods using quick score analysis; [d]$\chi^2$ test.

 © 2001 Cancer Research Campaign

The above features of the tumours in the high $G_2$RS cohort are distinct from those associated with other hereditary forms of breast cancer notably those accompanying germline mutations in BRCA1 or BRCA2. However, the characteristics of the non BRCA1 / BRCA2 familial tumours reported recently are similar to those found by us in the high $G_2$RS group (Lakhani et al, 2000). The non *BRCA1* / *BRCA2* tumours were of lower grade indicating presumably a more favourable prognosis.

Only 3 tumours in the series were of 'special type' (2 lobular carcinomas and 1 tubulo-lobular carcinoma). These tend to carry a relatively good prognosis. All 3 were in the high $G_2$RS group. One previous report found no significant difference in $G_2$RS according to tumour grade (Scott et al, 1999). However there was also some evidence that patients with larger tumours (T2 compared to T1) had higher $G_2$ scores (Scott et al, 1999) which is contrary to our findings. That study was not designed specifically to examine the issue of tumour characteristics and detailed analysis of the findings was not reported.

The possible mechanisms underlying $G_2$RS and the identity of the low penetrance genes involved in cancer predisposition have not yet been established but it seems likely that chromatid breaks arise by a process involving genomic rearrangements (Bryant, 1998; Rogers-Bald et al, 2000). The cellular response to DNA damage is regulated by a network of proteins. Following the induction of double-stranded breaks in chromosomal DNA, a complex network is activated that regulates DNA repair and progression through the cell cycle (Wang, 2000). Genes in this network protect the integrity of the genome and mutational events in these genes can predispose individuals to cancer. According to one scheme, cancer susceptibility genes can be conveniently classified into two groups: gatekeepers and caretakers (Kinzler and Vogelstein, 1997). Gatekeeper genes are important in cell cycle control and apoptosis. Caretaker genes are involved in DNA repair and inactivation results in genetic instability. We hypothesise that inactivation of caretaker genes influences the processing of DNA damage and thus leads to increased chromatid damage (high $G_2$RS). The low penetrance gene/s involved in predisposition to breast cancer might thus be members of the caretaker gene family.

The differences that we have reported in this preliminary study of patients with breast cancers in low and high $G_2$RS groups, would warrant a more detailed study on a larger cohort of breast cancer patients to determine whether the high $G_2$RS group do indeed have an improved prognosis and comprise a distinct subset for whom specific screening and management policies may be appropriate. Our observations may also facilitate the identification of the low penetrance genes involved. It would not be possible to use the $G_2$ assay as a population screening method for breast cancer as it is technically demanding and time-consuming. However if these low penetrance genes can be identified, then it will be possible to determine whether specific polymorphisms serve as markers for increased risk of breast cancer.

### ACKNOWLEDGEMENTS

We thank all patients who participated in the study and the Department of Health (A3.4 / RRX40) for funding the research.

### REFERENCES

Balslev I, Axelsson CK, Zedeler K, Rasmussen BB, Carstensen B and Mouridsen HT (1994) The Nottingham Prognostic Index applied to 9149 patients from the studies of the Danish Breast Cancer Co-operative Group. *Breast Cancer Res Treat* **32**: 281–290

Brown JM, Benson EA and Jones M (1993) Confirmation of a long-term prognostic index in breast cancer. *Breast* **2**: 144–147

Bryant PE (1998) The signal model: a possible explanation for the conversion of DNA double-strand breaks into chromatid breaks. *Int J Radiat Biol* **73**: 243–251

Chen PL, Sellers TA, Rich SS, Potter JD and Folsom AR (1995) Segregation analysis of breast cancer in a population-based sample of postmenopausal probands. *Genet Epidemiol* **12**: 401–415

Galea MH, Blamey RW, Elston CE and Ellis IOI (1992) The Nottingham prognostic index in primary breast cancer. *Breast Cancer Res Treat* **22**: 207–219

Houlston RS and Peto J (1996) Genetics and the common cancers. In: *Genetic Predisposition to Cancer.* Eeles RA, Ponder BAJ, Easton DF et al (eds) pp 208–226. Chapman and Hall: London

Kinzler KW and Vogelstein B (1997) Gatekeepers and caretakers. *Nature* **386**: 761–763

Lakhani SR, Easton DF, Stratton MR and the Breast Cancer Linkage Consortium (1997) Pathology of familial breast cancer: differences between breast cancers in carriers of BRCA1 or BRCA2 mutations and sporadic cases. *Lancet* **349**: 1505–1510

Lakhani SR, Jacquemier J, Sloane JP, Gusterson BA, Anderson TJ, van de Vijver MJ, Farid LM, Venter D, Antoniou A, Storfer-Isser A, Smyth E, Steel CM, Haites N, Scott RJ, Goldgar D, Neuhausen S, Daly PA, Ormiston W, McManus R, Scherneck S, Ponder BA, Ford D, Peto J, Stoppa-Lyonnet D, Easton DF et al (1998) Multifactorial analysis of differences between sporadic breast cancers and cancers involving BRCA1 and BRCA2 mutations. *J Natl Cancer Inst* **90**: 1138–1145

Lakhani SR, Gusterson BA, Jacquemier J, Sloane JP, Anderson TJ, van de Vijver MJ, Venter D, Freeman A, Antoniou A, McGuffog L, Smyth E, Steel CM, Haites N, Scott RJ, Goldgar D, Neuhausen S, Daly PA, Ormiston W, McManus R, Scherneck S, Ponder BA, Futreal PA, Peto J, Stoppa-Lyonnet D, Bignon YJ and Stratton MR (2000) The pathology of familial breast cancer: histological features of cancers in families not attributable to mutations in BRCA1 or BRCA2. *Clin Cancer Res* **6**: 782–789

Parshad R, Price FM, Bohr VA, Cowans KH, Zujewski JA and Sanford KK (1996) Deficient DNA repair capacity, a predisposing factor in breast cancer. *Br J Cancer* **74**: 1–5

Patel RK, Trivedi AH, Arora DC, Bhatavdekar JM and Patel DD (1997) DNA repair proficiency in breast cancer patients and their first-degree relatives. *Int J Cancer* **73**: 20–24

Peto J, Collins N, Barfoot R, Seal S, Warren W, Rahman N, Easton DF, Evans C, Deacon J and Stratton M (1999) Prevalence of BRCA1 and BRCA2 gene mutations in patients with early-onset breast cancer. *J Natl Cancer Inst* **91**: 943–949

Roberts SA, Spreadborough AR, Bulman B, Barber JBP, Evans DGR and Scott D (1999) Heritability of cellular radiosensitivity: a marker of low-penetrance predisposition genes in breast cancer? *Am J Hum Genet* **65**: 784–794

Rogers-Bald M, Sargent RG and Bryant PE (2000) Production of chromatid breaks by single dsb: evidence supporting the signal model. *Int J Radiat Biol* **76**: 23–29

Sanford KK, Parshad R, Gantt R, Tarone RE, Jones GM and Price FM (1989) Factors affecting and significance of $G_2$ chromatin radiosensitivity in predisposition to cancer. *Int J Radiat Biol* **55**: 963–981

Scott D, Spreadborough A, Levine E, Levine E and Roberts SA (1994) Genetic predisposition in breast cancer. *Lancet* **344**: 1444

Scott D, Spreadborough AR, Jones LA, Roberts SA and Moore CJ (1996) Chromosomal radiosensitivity in $G_2$-phase lymphocytes as an indicator of cancer predisposition. *Radiat Res* **145**: 3–16

Scott D, Barber JBP, Levine EL, Burrill W and Roberts SA (1998) Radiation-induced micronucleus induction in lymphocytes identifies a high frequency of radiosensitive cases among breast cancer patients: a test for predisposition. *Br J Cancer* **77**: 614–620

Scott D, Barber JBP, Spreadborough AR, Burrill W and Roberts SA (1999) Increased chromosomal radiosensitivity in breast cancer patients: a comparison of two assays. *Int J Radiat Biol* **75**: 1–10

Taylor AR (1983) The effects of radiation on the chromosomes of patients with an unusual cancer susceptibility. In: *Radiation induced chromosome damage in man*, Ishihar T, Sasaki M (eds) pp 167–199. Alan R Liss: New York

Teare MD, Wallace JA, Harris M, Howell A and Birch JM (1994) Cancer experience in the relatives of an unselected series of breast cancer patients. *Br J Cancer* **10**: 102–111

Terzoudi GI, Jung T, Hain J, Vrouvas J, Margaritis K, Donta-Bakoyianni C, Makropoulos V, Angelakis PH and Pantellas GE (2000) Increased $G_2$ chromosomal radiosensitivity in cancer patients: the role of cdk 1/cyclin-B activity level in the mechanisms involved. *Int J Radiat Biol* **76**: 607–615

Wang JYL (2000) New link in a web of human genes. *Nature* **405**: 404–405

*© 2001 Cancer Research Campaign* 
